# Haemoglobin, magnetic resonance imaging markers and cognition: a subsample of population-based study

**DOI:** 10.1186/s13195-018-0440-5

**Published:** 2018-11-06

**Authors:** Bryce Tan, Narayanaswamy Venketasubramanian, Henri Vrooman, Ching-Yu Cheng, Tien Yin Wong, Christopher Chen, Saima Hilal

**Affiliations:** 1 Memory Ageing and Cognition Centre (MACC), National University Health System, Singapore, Singapore; 2 Raffles Neuroscience Centre, Raffles Hospital, Singapore, Singapore; 3 Departments of Radiology & Medical Informatics, Erasmus University Medical Centre, Rotterdam, The Netherlands; 4 Singapore Eye Research Institute, Singapore National Eye Centre, Singapore, Singapore; 5 Academic Medicine Research Institute, Duke-NUS Graduate Medical School, Singapore, Singapore; 6 Department of Pharmacology, Yong Loo Lin School of Medicine, National University of Singapore, Level 4, Block MD3, 16 Medical Drive, Singapore, 117600 Singapore; 7 Departments of Epidemiology and Radiology and Nuclear Medicine, Erasmus University Medical Centre, Rotterdam, the Netherlands

**Keywords:** Haemoglobin, Anaemia, Cortical thinning, Microbleed, Cognitive impairment

## Abstract

**Background:**

Low haemoglobin is highly prevalent among the elderly and has been associated with dementia. However, the mechanisms underlying this association with cognitive dysfunction, either through cerebrovascular disease or neurodegeneration, remain poorly understood. We aimed to examine the association of decreased haemoglobin levels with markers of cerebral small vessel disease (CSVD), neurodegeneration and cognitive impairment in an elderly Asian population.

**Methods:**

A total of 796 Chinese, Malay and Indian participants aged 60 years and older from the Epidemiology of Dementia in Singapore study were included in this study. After providing information on demographics, anthropometry and cardiovascular risk factors, participants underwent 3-T brain magnetic resonance imaging (MRI) to measure markers of CSVD, including cerebral microbleeds, cortical cerebral microinfarcts, lacunes, enlarged perivascular spaces and white matter hyperintensities, as well as neurodegenerative markers, including cortical thickness and subcortical structure volumes quantified using FreeSurfer. Cognition was assessed using a detailed neuropsychological assessment. Logistic and linear regression models were constructed, adjusting for age, gender, education, race, body mass index, smoking, hypertension, hyperlipidaemia, diabetes, glomerular filtration rate and other MRI markers, to test the association between haemoglobin levels and the MRI markers and cognition.

**Results:**

Decreased haemoglobin levels were associated with cerebral microbleeds, specifically lobar microbleeds (OR, 1.21; 95% CI, 1.04–1.40; *p* = 0.015). Decreased haemoglobin levels were also associated with occipital cortical thinning (mean difference, − 0.011; 95% CI, − 0.019, − 0.004; *p* = 0.003) and smaller accumbens volume (mean difference, − 0.01; 95% CI, − 0.02, 0.00; *p* = 0.005). A significant association was also observed between decreased haemoglobin levels and poorer global cognitive performance (mean difference, − 0.04; 95% CI, − 0.09, 0.00; *p* = 0.048). In cognitive domain analysis, associations were again observed between decreased haemoglobin levels and worse performance on attention (mean difference, − 0.05; 95% CI, − 0.10, − 0.01; *p* = 0.028) and language (mean difference, − 0.06; 95% CI, − 0.12, 0.00; *p* = 0.048) domains; however, these associations did not survive multiple comparison.

**Conclusions:**

Decreased haemoglobin levels were associated with lobar microbleeds, neurodegenerative markers and cognitive dysfunction. Future studies should ascertain whether iron, folate or vitamin B_12_ supplementation is able to ameliorate the onset and progression of cognitive impairment and dementia associated with low haemoglobin.

## Background

There is growing evidence that anaemia is a common condition in the elderly; it has a prevalence exceeding 10% in people aged 65 years or older [1]. About one-third of anaemia diagnoses in the elderly are attributed to nutritional deficits which are easily treatable by adequate nutritional support with iron, vitamin B_12_ and folate supplements [2]. Recently, it has been suggested that even modest decreases in haemoglobin concentrations among those not classified as anaemic are associated with increased morbidity and mortality [3–5]. Although previous studies have shown that decreased haemoglobin levels are associated with cognitive impairment [6] and risk of dementia [7–9], these associations are inconsistent [10].

Non-invasive magnetic resonance imaging (MRI) markers of cerebral small vessel disease (CSVD) include lacunes, white matter hyperintensities, cerebral microbleeds and cortical cerebral microinfarcts, whereas surrogate markers of neurodegeneration include cortical and subcortical atrophy. Limited data has shown that anaemia is associated with a chronic hypoxic state that contributes to increased cerebrovascular burden, resulting in increased white matter hyperintensity volume [11–13]. However, a recent study showed no association between lower haemoglobin levels and the presence or progression of CSVD [14]. Moreover, the association of haemoglobin with cerebral microbleeds, and cortical cerebral microinfarcts remain unstudied. With respect to neurodegenerative markers, only one study reported that lower haemoglobin levels are linked to cortical thinning among cognitively normal women [14]. These differences in results may be attributed to heterogeneity in defining anaemia (binary criteria vs. percentiles) or small sample sizes, or they may be related to the study of particular disease populations (dementia, chronic kidney disease and hypertension). Hence studies from a general population are needed to investigate the relationship between decreased haemoglobin levels and markers of CSVD and neurodegeneration to investigate the underlying mechanisms. Such findings would have potential clinical significance for preventing cognitive impairment and improving cognitive function in the elderly.

We aimed to examine the association of haemoglobin with markers of CSVD (cerebral microbleeds, cortical cerebral microinfarcts, lacunes, enlarged perivascular spaces and white matter hyperintensities) and neurodegeneration (cortical thickness and subcortical structure volume). We also examined the effects of decreased haemoglobin levels on cognitive performance in a multi-ethnic Asian population. We hypothesize that decreased haemoglobin levels induce a hypoxic state leading to ischemia as well as atrophy in subcortical and cortical regions.

## Methods

### Study population

The Epidemiology of Dementia in Singapore study recruited individuals from the Singapore Epidemiology of Eye Disease study, which comprised participants between 40 and 85 years old who participated in the Singapore Chinese Eye Study, the Singapore Malay Eye Study and the Singapore Indian Eye Study [15]. Briefly, participants aged 60 years and older were screened with the Abbreviated Mental Test (AMT) and a self-reported history of forgetfulness. Screen-positives were defined on the basis of education-based cut-offs on AMT (AMT ≤ 6 in subjects with ≤ 6 years of formal education or ≤ 8 among those with > 6 years of formal education) or if the caregiver confirmed progressive forgetfulness. Thus the inclusion criteria of the study included (1) screen-positive on AMT or Present Functioning Questionnaire and (2) written informed consent given by participants or their legally acceptable representatives. Of these 1598 screen-positive participants, 957 agreed to participate in the second phase, which included brain MRI and extensive cognitive assessment. Exclusion criteria included participant or legally acceptable representative not willing to provide written informed consent. Ethics approval was obtained from the Singapore Eye Research Institute and the National Healthcare Group domain-specific review board, and written consent was obtained from participants.

### Blood tests

Participants were required to fast for at least 8 h prior to blood tests. Fasting blood samples were sent to the National University Hospital Laboratory for measurements of the following: full blood count, glucose, lipid panel and creatinine. Haemoglobin was measured with the Sysmex XN-Series automated haematology analyser 2012 version (Streck, Omaha, NE, USA). The lipid panel comprised total cholesterol, low-density lipoprotein, high-density lipoprotein and triglyceride levels.

### Neuroimaging

MRI scans were obtained using a 3-T MAGNETOM Trio Tim scanner (Siemens, Erlangen, Germany) with a 32-channel head coil at the Clinical Imaging Research Centre, National University of Singapore. Subjects with claustrophobia, with contraindications for MRI, or who were unable to tolerate the procedure were excluded. STRIVE (Standards for Reporting Vascular Changes on Neuroimaging) criteria were used for grading of lacunes on fluid-attenuated inversion recovery (FLAIR) and T2-weighted sequences [16]. Cortical cerebral microinfarcts were graded on FLAIR, T1- and T2-weighted images and were defined as hypointense lesions on T1-weighted images, restricted to the cortex, < 5 mm in diameter, and perpendicular to the cortical surface. These lesions were further confirmed as hyperintense or isointense on T2-weighted and FLAIR images as previously described [17] and were analysed as a categorical variable. Cerebral microbleeds were graded using the Brain Observer Microbleed Scale [18] and are classified as lobar or deep microbleeds as dichotomous variables. Enlarged perivascular spaces were categorized as ≤ 10 vs. ≥ 11 to compare moderate to severe enlarged perivascular spaces with mild to absent perivascular spaces [19].

Total intracranial volume was quantified using T1- and T2-weighted images, whereas white matter hyperintensity volume was quantified with FLAIR images using automatic segmentation at the Erasmus University Medical Centre, the Netherlands. A model-based automated procedure (FreeSurfer version 5.1.0) was used for measurements of cortical thickness and subcortical structure volumes on T1-weighted images. Briefly, cortical thickness was measured by taking the shortest distance between the white–grey matter boundary and pial surface for regional lobes and the whole brain at each vertex. Cortical thickness of the frontal, insular, limbic, occipital, parietal and temporal regions was measured, and a parcellation guide on the gyral and sulcal structures of the cerebral cortex was used in calculating the average cortical thickness of the left and right lobes [20]. Volumes of subcortical structures of each hemisphere (accumbens, amygdala, brainstem, caudate, hippocampus, putamen, pallidum and thalamus) were calculated by segmentation using rigid-body registration and subjected to non-linear normalization with respect to a probabilistic brain atlas [21].

### Covariate assessment

Demographic and cardiovascular risk factors were collected during the interview and included age, gender, education, smoking history, height, weight, hypertension, hyperlipidaemia and diabetes mellitus. A digital automatic blood pressure machine (HEM-7203; OMRON, Kyoto, Japan) was used for measurement of two systolic and diastolic blood pressure readings taken 5 min apart after the participant had rested for 5 min. Hypertension was defined as the use of anti-hypertensive medication or systolic blood pressure ≥ 140 mmHg and/or diastolic blood pressure ≥ 90 mmHg. Diabetes mellitus was defined as the use of diabetic medications or a glycosylated haemoglobin ≥ 6.5%. Hyperlipidaemia was defined as the use of lipid-lowering medications or total cholesterol level ≥ 4.14 mmol/L. Education was categorized as ≤ 6 years or > 6 years of formal education. Body mass index (BMI) was calculated by weight in kilograms divided by height in meters squared. Smoking was categorized into ever vs. never smokers. Socio-economic status was defined by monthly income and housing. A low socio-economic status was categorized by monthly income < 2000 Singapore dollars per household and living in ≤ 2-room public housing flats.

### Cognitive assessment

All participants underwent detailed cognitive assessment using validated methods [15]. The following seven domains were tested: attention, executive function, language, visual memory, verbal memory, visuomotor speed and visuoconstruction. Each domain was tested using the following cognitive tests:

- Attention: Digit Span, Visual Memory Span [22] and Auditory Detection [23]
- Executive function: Frontal Assessment Battery [24] and Maze Task [25]
- Language: Boston Naming Test [26] and Verbal Fluency [27]
- Verbal memory: Word List Recall [28] and Story Recall
- Visual memory: Picture Recall, Wechsler Memory Scale–Revised (WMS-R) Visual Reproduction [22]
- Visuoconstruction: WMS-R Visual Reproduction Copy Task [22], Clock Drawing [29], and Wechsler Adult Intelligence Scale–Revised subtest of block design [30]
- Visuomotor speed: Symbol Digit Modality Test [31] and Digit Cancellation [32].

For each participant, raw scores from each individual test within a domain were first transformed to standardized Z-scores using the mean and SD of that test in this cohort. Subsequently, for each participant a mean Z-score for each domain was calculated by averaging the Z-scores of all the individual tests within that domain. These mean Z-scores of each domain were then standardized using the mean and SD of that domain-specific Z-score. Finally, a composite Z-score reflecting global cognitive functioning was calculated by averaging the seven domain-specific mean Z-scores, which were also standardized using the corresponding mean and SD.

### Statistical Analysis

Comparisons between included and excluded participants were performed using the chi-square test for categorical variables and Student’s *t* test for continuous variables. Binary logistic regression models with ORs and 95% CIs were constructed to determine the association of haemoglobin with microbleeds, cortical cerebral microinfarcts, lacunes and enlarged perivascular spaces, whereas linear regression models were used to determine the association between haemoglobin and white matter hyperintensity volumes with mean differences and 95% CIs. Linear regression models were again constructed to determine the association of haemoglobin with global and regional cortical thickness, subcortical structural volume, and cognitive domains. Models were initially adjusted for age, gender, race, smoking status and education (in analysis with cognition) (model I). Further adjustments were made for vascular risk factors, which included diabetes mellitus, hyperlipidaemia, hypertension, glomerular filtration rate and BMI (model II). In the fully adjusted model (model III), socio-economic status (in analysis with cognition) and MRI markers (microbleeds, cortical cerebral microinfarcts, lacunes, enlarged perivascular spaces, white matter hyperintensity volume, microbleeds) were included to investigate the independent effects of haemoglobin on cortical thickness, subcortical structure volume, and cognition. Mean differences or ORs, 95% CIs and their corresponding *p* values in the tables correspond to the effect estimate for each gram per decilitre drop in haemoglobin. *p* values < 0.05 were considered significant. In view of the multiple testing performed in cortical thickness, subcortical structures and cognition models, the Bonferroni correction was applied with the significance levels set at *p* = 0.025 (0.05/2) for CSVD, *p* = 0.0083 (0.05/6) for cortical thickness, *p* = 0.00625 (0.05/8) for subcortical structures and *p* = 0.0071 (0.05/7) for cognition. All statistical analyses were performed using standard statistical software (IBM SPSS Statistics version 24; IBM, Armonk, NY, USA).

## Results

Assessment of subjects was performed from August 12, 2010, to July 24, 2015. Among 957 subjects, 46 were diagnosed with dementia, 96 had ungradable MRI scans and 19 had no haemoglobin results available. The final sample consisted of 796 subjects. Table 1 presents the baseline data of the included (*n* = 796) and excluded (*n* = 756) subjects. The excluded group consisted of positive-screened non-responders, persons with ungradable MRI scans or absent haemoglobin levels. Briefly, excluded subjects were older, more likely to be female, to be less educated and to have hypertension and less likely to have hyperlipidaemia.

**Table 1:** Baseline characteristics of included and excluded subjects

	Included (*n* = 796)	Excluded (*n* = 756)^a^	*p* Value
Age, years, mean (SD)	70.0 (6.6)	71.9 (6.9)	< 0.001
Female sex, *n* (%)	432 (54.3)	433 (57.3)	0.015
Education (≤ 6 years), *n* (%)	489 (61.4)	554 (73.3)	< 0.001
Ethnicity			< 0.001
Chinese, *n* (%)	273 (34.2)	333 (44.0)	
Malay, *n* (%)	263 (33.0)	194 (25.7)	
Indian, *n* (%)	260 (32.7)	229 (30.3)	
Diabetes mellitus, *n* (%)	289 (36.3)	260 (34.4)	0.430
Hypertension, *n* (%)	621 (78.0)	634 (83.9)	0.003
Hyperlipidaemia, *n* (%)	586 (73.6)	498 (65.9)	0.001
Smoking, *n* (%)	198 (24.9)	185 (24.4)	0.854
BMI, kg/m^2^, mean (SD)	23.4 (4.6)	23.6 (4.6)	0.982
Mean arterial blood pressure, mmHg, mean (SD)	97.4 (10.4)	97.8 (11.3)	0.282
Total cholesterol, mmol/L, mean (SD)	5.1 (1.2)	5.2 (1.2)	0.354
Random blood glucose, mmol/L, mean (SD)	7.1 (3.1)	7.1 (3.1)	0.846
Haemoglobin, g/dl, mean (SD)	13.4 (1.4)	–	–
Creatinine, μmol/L, median (IQR)	72 (30)	–	–
eGFR, ml/min, mean (SD)	91 (29)	–	–

*BMI* Body mass index, *eGFR* Estimated glomerular filtration rate

^a^Excluded subjects were screened positive non-responders, had ungradable magnetic resonance imaging scans or absent haemoglobin levels

Table 2 presents the association of haemoglobin with MRI markers of CSVD. Decreased levels of haemoglobin were associated with cerebral microbleeds (OR, 1.16; 95% CI, 1.01–1.33), specifically those located in the lobar region (OR, 1.21; 95% CI, 1.04–1.40) in the multivariable model (even after adjusting for multiple comparisons). Although decreased levels of haemoglobin were associated with lacunes (OR, 1.25; 95% CI, 1.07–1.46) in the models adjusted for age, gender, race and smoking, the association became attenuated in the presence of cardiovascular risk factors and other MRI markers of CSVD.

**Table 2:** Associations of haemoglobin with markers of cerebral small vessel disease

Hb (per g/dl decrease)	Cerebral microbleeds			Cortical cerebral microinfarcts	Lacunes	Enlarged perivascular spaces^a^	WMH volume (ml)
	All microbleeds OR (95% CI)^b^	Lobar microbleeds OR (95% CI)^b^	Deep microbleeds OR (95% CI)^b^	OR (95% CI)^b^	OR (95% CI)^b^	OR (95% CI)^b^	β (95% CI)^b^
Model I	1.16 (1.02, 1.31)	1.18 (1.04, 1.34)	1.06 (0.90, 1.25)	1.04 (0.82, 1.33)	1.25 (1.07, 1.46)	1.06 (0.91, 1.26)	0.10 (−1.25, 1.44)
	*p* = 0.019	*p* = 0.011	*p* = 0.481	*p* = 0.736	*p* = 0.004	0.458	*p* = 0.889
Model II	1.18 (1.03, 1.36)	1.23 (1.06, 1.43)	1.07 (0.88, 1.29)	0.93 (0.72, 1.21)	1.12 (0.94, 1.34)	1.06 (0.89, 1.27)	0.35 (−0.07, 0.76)
	*p* = 0.017	*p* = 0.006	*p* = 0.512	*p* = 0.602	*p* = 0.198	0.489	*p* = 0.102
Model III	1.16 (1.01, 1.33)	1.21 (1.04, 1.40)	1.03 (0.85, 1.25)	0.92 (0.70, 1.20)	1.07 (0.89, 1.29)	1.05 (0.88, 1.26)	−0.21 (− 0.19, 0.60)
	*p* = 0.039	*p* = 0.015^c^	*p* = 0.754	*p* = 0.629	*p* = 0.467	0.579	*p* = 0.299

*Hb* Haemoglobin, *WMH* White matter hyperintensity

Model I: adjusted for age, gender, race and smoking status

Model II: Model I + hypertension, hyperlipidaemia, diabetes mellitus, glomerular filtration rate and body mass index

Model III: Model II + other magnetic resonance imaging markers

^a^Present in a subsample of 536 persons and categorized as ≤ 10 vs. ≥ 11

^b^The reported CIs and *p* values are for decreased haemoglobin levels. These values are extracted from the multivariable models that also included covariates

^c^Significant after Bonferroni correction *p* < 0.025

Table 3 presents the associations of haemoglobin with global and regional cortical thickness. Decreased haemoglobin levels were associated with smaller global (mean difference, -0.006, 95% CI: -0.013; − 0.001) and occipital (mean difference, − 0.012; 95% CI, − 0.020, − 0.005) cortical thickness after controlling for demographic and cardiovascular risk factors. These associations remained significant after additional adjustment for markers of CSVD and intracranial volume. When we applied the Bonferroni correction, decreased haemoglobin levels remained significantly linked with occipital cortical thinning. With respect to the subcortical structures, decreased levels of haemoglobin were associated with smaller accumbens volume (mean difference, − 0.02; 95% CI, − 0.03, − 0.01) in the demographic and cardiovascular risk factors model. These associations remained unaltered after controlling for MRI markers and survived multiple testing. No association was observed with other subcortical structure volumes (Table 4).

**Table 3:** Association of haemoglobin with global and regional cortical thickness

Hb (per g/dl decrease)	Global cortical thickness (mm), β (95% CI)^a^	Region-specific cortical thickness					
		Frontal (mm) β (95% CI)*	Insula (mm) β (95% CI)*	Occipital (mm) β (95% CI)*	Temporal (mm) β (95% CI)*	Limbic (mm) β (95% CI)*	Parietal (mm) β (95% CI)*
Model I	− 0.009 (− 0.014, − 0.003)	−0.006 (− 0.012, − 0.001)	−0.007 (− 0.015, 0.001)	−0.015 (− 0.022, − 0.008)	−0.009 (− 0.016, − 0.002)	−0.007 (− 0.013, − 0.001)	−0.009 (− 0.016, − 0.002)
	*p* = 0.002	*p* = 0.032	*p* = 0.105	*p* < 0.001	*p* = 0.014	*p* = 0.015	*p* = 0.010
Model II	−0.006 (− 0.013, − 0.001)	−0.005 (− 0.011, 0.002)	−0.004 (− 0.013, 0.005)	−0.012 (− 0.020, − 0.005)	−0.007 (− 0.014, 0.000)	−0.006 (− 0.012, 0.001)	−0.005 (− 0.013, 0.002)
	*p* = 0.025	*p* = 0.149	*p* = 0.426	*p* = 0.001	*p* = 0.066	*p* = 0.076	*p* = 0.185
Model III	− 0.005 (− 0.011, 0.001)	− 0.003 (− 0.009, 0.003)	−0.001 (− 0.010, 0.008)	−0.011(− 0.019, − 0.004)	−0.005 (− 0.012, 0.003)	−0.004 (− 0.020, 0.012)	− 0.004 (− 0.011, 0.004)
	*p* = 0.091	*p* = 0.299	*p* = 0.814	*p* = 0.003^b^	*p* = 0.219	*p* = 0.185	*p* = 0.320

*Hb* haemoglobin

Model I: adjusted for age, gender, race and smoking status

Model II: Model I + hypertension, hyperlipidaemia, diabetes mellitus, glomerular filtration rate and BMI

Model III: Model II + magnetic resonance imaging markers (intracranial volume, lacunes, white matter hyperintensities, cerebral microbleeds, cortical cerebral microinfarcts)

^a^The reported confidence interval and *p* values are for decreased haemoglobin levels. These values are extracted from the multivariable models which also included covariates

^b^Significant after Bonferroni correction: *p* = 0.0083

**Table 4:** Association of haemoglobin with subcortical structure volumes

Hb (per g/dl decrease)	Accumbens (ml), β (95% CI)^a^	Amygdala (ml), β (95% CI)^a^	Caudate (ml), β (95% CI)^a^	Pallidum (ml), β (95% CI)^a^	Putamen (ml), β (95% CI)^a^	Thalamus (ml), β (95% CI)^a^	Hippocampus (ml), β (95% CI)^a^	Brainstem (ml), β (95% CI)^a^
Model I	− 0.02 (− 0.03, − 0.01)	−0.02 (− 0.04, 0.00)	−0.03 (− 0.08, 0.02)	−0.01 (− 0.04, 0.01)	−0.08 (− 0.14, − 0.01)	−0.03 (− 0.09, 0.02)	−0.04 (− 0.08, 0.01)	−0.15 (− 0.27, − 0.03)
	*p* < 0.001	*p* = 0.055	*p* = 0.291	*p* = 0.233	*p* = 0.019	*p* = 0.245	*p* = 0.097	*p* = 0.014
Model II	−0.02 (− 0.03, − 0.01)	−0.02 (− 0.04, 0.01)	0.00 (− 0.06, 0.05)	0.00 (− 0.03, 0.02)	−0.05 (− 0.11, 0.02)	−0.01 (− 0.06, 0.07)	−0.02 (− 0.07, 0.02)	−0.07 (− 0.20, 0.06)
	*p* = 0.001	*p* = 0.134	*p* = 0.899	*p* = 0.833	*p* = 0.190	*p* = 0.870	*p* = 0.366	*p* = 0.312
Model III	−0.01 (−0.02, 0.00)	−0.01 (− 0.04, 0.01)	−0.02 (− 0.07, 0.03)	0.00 (− 0.02, 0.02)	−0.05 (− 0.12, 0.02)	0.01 (− 0.04, 0.07)	−0.02 (− 0.06, 0.03)	−/0.04 (− 0.16, 0.08)
	*p* = 0.005^b^	*p* = 0.215	*p* = 0.493	*p* = 0.912	*p* = 0.169	*p* = 0.629	*p* = 0.433	*p* = 0.551

*Hb* Haemoglobin

Model I: adjusted for age, gender, race and smoking status

Model II: Model I + hypertension, hyperlipidaemia, diabetes mellitus, glomerular filtration rate and body mass index

Model III: Model II + magnetic resonance imaging markers (intracranial volume, lacunes, white matter hyperintensities, cerebral microbleeds, cortical cerebral microinfarcts)

^a^The reported confidence interval and *p* values are for decreased haemoglobin levels. These values are extracted from the multivariable models which also included covariates

^b^Significant after Bonferroni correction *p* = 0.00625

Decreased levels of haemoglobin were also significantly associated with poor cognitive performance (mean difference in global cognitive z-scores, − 0.06; 95% CI, − 0.10, − 0.01) as well as with individual cognitive domains of attention (mean difference, − 0.06; 95% CI, − 0.11, − 0.01) and language (mean difference, − 0.07; 95% CI, − 0.13, − 0.02) after adjustment for demographic and cardiovascular risk factors. Adjustment for socio-economic status and other MRI markers of CSVD rendered similar results, suggesting that the associations of haemoglobin with cognition were not mediated by these variables (Table 5). However, these associations did not reach the revised level of significance after the Bonferroni correction was applied.

**Table 5:** Association of haemoglobin levels with cognition

Hb (per g/dl decrease)	Global cognition	Domain specific cognitive performance						
	β (95% CI)^a^	Executive β (95% CI)^a^	Attention β (95% CI)^a^	Language β (95% CI)^a^	Verbal Memory β (95% CI)^a^	Visual Memory β (95% CI)^a^	Visuoconstruction β (95% CI)^a^	Visuomotor speed β (95% CI)^a^
Model I	−0.05 (− 0.10, − 0.01)	−0.06 (− 0.11, − 0.01)	−0.05 (− 0.09, 0.00)	−0.07 (− 0.12, − 0.02)	−0.02 (− 0.06, 0.03)	−0.05 (− 0.09, 0.00)	−0.04 (− 0.09, 0.00)	−0.04 (− 0.08, 0.00)
	*p* = 0.011	*p* = 0.017	*p* = 0.041	*p* = 0.007	*p* = 0.457	*p* = 0.039	*p* = 0.055	*p* = 0.069
Model II	−0.06 (−0.10, − 0.01)	−0.06 (− 0.11, − 0.01)	−0.06 (− 0.11, − 0.01)	−0.07 (− 0.12, − 0.02)	−0.01 (− 0.06, 0.04)	−0.05 (− 0.10, 0.00)	−0.04 (− 0.09, 0.01)	−0.03 (− 0.08, 0.01)
	*p* = 0.014	*p* = 0.020	*p* = 0.014	*p* = 0.012	*p* = 0.666	*p* = 0.063	*p* = 0.081	*p* = 0.132
Model III	−0.04 (−0.09, 0.00)	−0.05 (− 0.10, 0.01)	−0.05 (− 0.10, − 0.01)	−0.06 (− 0.12, 0.00)	−0.01 (− 0.06, 0.05)	−0.04 (− 0.09, 0.01)	−0.03 (− 0.08, 0.02)	−0.03 (− 0.07, 0.02)
	*p* = 0.048	*p* = 0.078	*p* = 0.028	*p* = 0.048	*p* = 0.395	*p* = 0.143	*p* = 0.176	*p* = 0.217

*Hb* Haemoglobin

Model I: adjusted for age, gender, race, education and smoking status

Model II: Model I + hypertension, hyperlipidaemia, diabetes mellitus, glomerular filtration rate and body mass index

Model III: Model II + socio-economic status + magnetic resonance imaging markers (intracranial volume, lacunes, white matter hyperintensities, cerebral microbleeds, cortical cerebral microinfarct)

^a^The reported CIs and *p* values are for decreased haemoglobin levels. These values are extracted from the multivariable models which also included covariates

None of the associations reached revised statistical significance (Bonferroni-corrected *p* = 0.0071)

## Discussion

Our results showed that decreased levels of haemoglobin were associated with lobar microbleeds, global and occipital cortical thinning, and smaller accumbens volume independent of cardiovascular risk factors and other MRI markers. Moreover, people with decreased haemoglobin levels had worse cognition specifically in the domains of attention and language, albeit these results were non-significant after correcting for multiple comparisons.

Few studies have explored the link between decreased haemoglobin levels and neuroimaging markers of CSVD, and the results remain controversial. One study has reported a link between anaemia and white matter hyperintensity progression among patients with hypertension [13], whereas a recent study failed to find an association with white matter hyperintensity volume in a large community-based study [14]. The latter study also did not find any significant link with lacunes and cerebral microbleeds [14]. Although we observed a significant association with lacunes in the initial model, the association was attenuated after adjustment for other markers of CSVD. Similarly, we did not observe any significant association with white matter hyperintensity volumes, which is consistent with the previous data. To date, this is the first study exploring the association between haemoglobin and enlarged perivascular spaces. Consistent with lacunes and white matter hyperintensities, we also did not observe an association with enlarged perivascular spaces, suggesting a similar underlying mechanism for the development of these lesions. On the contrary, we report an association of decreased haemoglobin levels with cerebral microbleeds, specifically with those located in the lobar region, which may reflect subclinical cerebral amyloid angiopathy. It has been suggested that prolonged decreased concentrations of haemoglobin may contribute to microvascular damage [33] and that they exacerbate cerebral ischemia by creating a state of chronic cerebral hypoxia [34]. This hypoxic state increases amyloid-β 1–42 levels via upregulation of β-secretase cleavage of amyloid precursor protein and β-secretase enzyme [35, 36], which promote amyloid plaque formation and contribute to neuronal death. In addition, cerebral microvascular smooth muscle cell damage resulting from hypoxic conditions further enhances amyloid angiopathy [37] and exacerbates chronic cerebral hypoxia [38].

With regard to neurodegenerative markers, only one previous study reported an association between decreased haemoglobin levels and cortical thinning in the frontal and parietal-temporal-occipital lobes in women [14]. Our results support previous findings by reporting associations between decreased haemoglobin levels and occipital cortical thinning. Furthermore, the effect estimates observed are consistent in both studies for the occipital lobe (β = − 0.011 mm; 95% CI, − 0.019 to − 0.004 mm; compared with β = − 0.011 mm; 95% CI, − 0.019 to − 0.003 mm) [14]. The chronic hypoxic state induced by decreased haemoglobin levels may accelerate cortical thinning and hence neurodegeneration. Moreover, as indicated above, chronic hypoxia may induce amyloidogenic processing, leading to brain atrophy. Interestingly, our study showed a consistent pattern in the association of decreased haemoglobin with microbleeds as well as with occipital thinning. Of note, the occipital cortex has the largest accumulation of cerebral amyloid angiopathy-related pathology on brain autopsy [39, 40]. The occipital lobe is susceptible to neurotoxic effects of amyloid [41, 42], and the extent of amyloid burden correlated with the severity of cortical thinning and metabolism in these regions [43]. Taken together, a chronic hypoxic state could contribute to vascular amyloid formation selectively in the occipital lobe as compared with other lobes. Concomitantly, we also observed a significant association of decreased haemoglobin with smaller accumbens volumes. The accumbens is more susceptible than other subcortical structures to amyloid deposition [44], and thus its vulnerability to hypoxia-mediated amyloid insults is increased.

Our study also found an association between decreased haemoglobin levels and poorer cognitive function specifically in the domains of attention and language. It has been suggested that cerebral hypoxia or reduced aerobic capacity as a consequence of chronic low haemoglobin levels may contribute to cognitive decline. It has also been suggested that the accumbens nucleus is more vulnerable to amyloid and tau deposition. Moreover, it is reported that the accumbens is directly connected to the medial temporal lobe, cingulate gyrus and precuneus, which are associated with language and learning and hence impairments in these domains. This is in line with a previous study which showed a link between decreased haemoglobin levels and verbal fluency, a task assessing language [6]. However, these associations in our study did not reach the revised level of significance after applying the Bonferroni correction. Nevertheless, the direction of the effect estimates does suggest that there is a link between decreased lower haemoglobin levels and cognitive dysfunction. It is noteworthy that most of the individuals in this study were cognitively normal or in the preclinical stages of dementia, where mild to moderate decrease in haemoglobin concentrations may have less effect on oxygen delivery to the brain through compensatory mechanisms such as vascular dilation to maintain cerebral blood flow and hence produce less deleterious effects on cognition. It is also possible that decreased haemoglobin levels may simply be a marker of chronic inflammation, frailty and declining health status, the conditions associated with cognitive impairment and dementia. Similarly, CSVD is associated with underlying neuroinflammatory response, and could reflect global inflammatory status [45, 46], which in turn could result in lower haemoglobin levels. However, the interactions between CSVD and haemoglobin remain poorly investigated and should be addressed in future studies.

Our results suggest that decreased haemoglobin levels may lead to measurable brain parenchymal damage. Hence, persons with low haemoglobin levels might benefit from iron, folate and vitamin B_12_ supplementation to improve cognitive performance. Although there have been previous clinical trials on exploring the beneficial effects of iron [47, 48], folate [49] and vitamin B_12_ [50–52] on reducing cognitive impairment, these studies have not yielded promising results. However, it remains possible that a subgroup of the population with anaemia could benefit from vitamin B or iron supplementation. This has not been investigated to date in post hoc analyses of these trials, and it could be a potential area for further research.

Strengths of our study include that it is a large multi-ethnic Asian population-based study; standardized and automated imaging techniques were used to measure cortical thickness, volumes of white matter hyperintensity and subcortical structures; and extensive neuropsychological assessment. Limitations of the study include the following:

1. Forty-nine percent of screened-positive subjects were not included in the second phase of the study. Compared with the included subjects, excluded subjects were older, more likely to be female, to be less educated and to have hypertension and less likely to have hyperlipidaemia, which might suggest that these excluded subjects were more likely to be cognitively impaired. Despite this non-participation, we found significant associations between decreased levels of haemoglobin and MRI markers and cognitive impairment, suggesting that the true effect estimates must have been larger.
2. Owing to the cross-sectional design of our study, we were unable to examine the temporal associations of haemoglobin with development of cognitive impairment, and incident MRI.
3. Microbleeds as detected on MRI may provide an indirect measure of amyloid deposition, and other imaging modalities such as amyloid positron emission tomography could allow more accurate measurement of total (vascular and parenchymal) amyloid deposition in the brain.
4. We did not exclude or adjust for several other chronic conditions, such as chronic kidney disease, or immunocompromised diseases, such as cancer or human immunodeficiency virus, which may have the potential to decrease haemoglobin levels in blood. However, because this is a subsample of a population-based study, the influence of such diseases is estimated to be minimal.

## Conclusions

Our study showed significant associations of decreased haemoglobin levels with lobar microbleeds, cortical thinning, accumbens atrophy and cognitive impairment in a large multi-ethnic Asian cohort. Because modest reduction in haemoglobin may be able to induce subtle brain changes and hence cognitive impairment, future studies should ascertain whether iron, folate or B_12_ supplementation is able to ameliorate the onset and progression of anaemia-associated cognitive impairment and dementia.

## Abbreviations

AMT: Abbreviated Mental Test
BMI: Body mass index
CSVD: Cerebral small vessel disease
eGFR: Estimated glomerular filtration rate
FLAIR: Fluid-attenuated inversion recovery
MRI: Magnetic resonance imaging
WMS-R: Wechsler Memory Scale–Revised
